# Comparison of the yields and properties of dedifferentiated fat cells and mesenchymal stem cells derived from infrapatellar fat pads

**DOI:** 10.1016/j.reth.2022.11.006

**Published:** 2022-11-29

**Authors:** Shunichi Fujii, Kentaro Endo, Seiya Matsuta, Keiichiro Komori, Ichiro Sekiya

**Affiliations:** Center for Stem Cell and Regenerative Medicine, Tokyo Medical and Dental University (TMDU), Tokyo, Japan

**Keywords:** Dedifferentiated fat cells, Mesenchymal stem cells, Infrapatellar fat pad, Osteoarthritis, Adipocytes, CPC, cetylpyridinium chloride, DFAT, dedifferentiated fat cells, EDTA, ethylenediaminetetraacetic acid, FBS, fetal bovine serum, GAG, glycosaminoglycan, IFP, infrapatellar fat pad, MSCs, mesenchymal stem cells, OA, osteoarthritis, PBS, phosphate-buffered saline, SC, subcutaneous fat tissue, SD, standard deviation, SVF, stromal vascular fraction

## Abstract

**Introduction:**

Infrapatellar fat pad (IFP)-derived mesenchymal stem cells (MSCs) have high chondrogenic potential and are attractive cell sources for cartilage regeneration. During ceiling culture to acquire the characteristics of MSCs, mature adipocytes from fat tissue are known to undergo dedifferentiation, generating dedifferentiated fat (DFAT) cells. The purpose of the present study was to compare the yields and biological properties of IFP-derived MSCs and IFP-derived DFAT cells.

**Methods:**

IFPs were harvested from the knees of 8 osteoarthritis (OA) patients. DFAT cells were obtained using a ceiling culture of adipocytes isolated from the floating top layer of IFP digestion. MSCs were obtained by culturing precipitated stromal vascular fraction cells. We compared the P0 cell yields, surface antigen profile, colony formation ability, and multipotency of DFAT cells and MSCs.

**Results:**

The P0 cell yields per flask and the estimated total cell yields from 1 g of IFP were much greater for MSCs than for DFAT cells. Both MSCs and DFAT cells were positive for MSC markers. No obvious difference was observed in colony formation ability. In differentiation assays, DFAT cells produced greater amounts of lipid droplets, calcified tissue, and glycosaminoglycan than MSCs did. Adipogenic and chondrogenic gene expressions were upregulated in DFAT cells.

**Conclusions:**

IFP-derived DFAT cells showed higher adipogenic and chondrogenic potentials than IFP-derived MSCs, but they had a poor cell yield.

## Highlights

• Yield of IFP-derived DFAT cells is much lower than that of IFP-derived MSCs.
• IFP-derived DFAT cells have greater chondrogenic capacity than IFP-derived MSCs.
• IFP-derived DFAT cells can be used in cartilage regeneration if the yield is higher.

## 1 Introduction

Articular cartilage consists of a few chondrocytes and an abundant extracellular matrix [1]. Articular cartilage has limited self-repairing ability due to its avascular nature. Untreated damaged cartilage often progresses to osteoarthritis (OA) [2]. Although autologous chondrocyte transplantation is one of the treatment options for cartilage defects, several issues remain unresolved, such as donor site morbidity and the dedifferentiation of cultured chondrocytes during culture [3]. New cell sources are required to treat injured cartilage.

Mesenchymal stem cells (MSCs) are promising cell sources for cartilage regenerative medicine. MSCs have high proliferative ability and multipotency to differentiate into adipocytes, osteoblasts, and chondrocytes [4]. MSCs can be isolated from various tissues, such as bone marrow, adipose tissue, and synovium. Subcutaneous fat tissue (SC)-derived MSCs are often used because they can be collected with minimally invasive procedures. However, the chondrogenic potential of SC-derived MSCs is inferior to that of bone marrow-, synovium-, and periosteum-derived MSCs [5,6].

The infrapatellar fat pad (IFP) is an adipose tissue located inside the knee and is mainly composed of mature adipocytes. During total knee arthroplasty, it is commonly resected and discarded [7]. The IFP serves as a reservoir for MSCs, which have higher proliferative ability and are more committed to chondrogenic lineage than SC-derived MSCs [8]. Given these helpful characteristics, IFP-derived MSCs are expected to be an attractive cell source for cartilage regeneration [9].

Dedifferentiated fat cells (DFAT cells) also hold great promise for cartilage regeneration. Mature adipocytes from SC are known to undergo dedifferentiation into fibroblast-like cells when cultured by the ceiling culture method [10,11]. DFAT cells acquire self-renewal and multi-differentiation abilities and exhibit characteristics similar to those of MSCs [12]. DFAT cells are considered to have relatively homogeneous cell populations compared with SC-derived MSCs, which leads to greater abilities both in vitro and in vivo [13,14]. In addition, the yields of primary DFAT cells from 1 g of SC tissue are much larger than those of primary SC-derived MSCs [12]. A recent study found that mature adipocytes from IFPs also underwent dedifferentiation under ceiling culture and that IFP-derived DFAT cells had higher chondrogenic differentiation capacity than SC-derived DFAT cells [15]. However, to our knowledge, no reports exist that compare the yields of IFP-derived DFAT cells and MSCs, and it remains unclear which cells have greater potential. The purpose of the present study was to compare the yields and biological properties, including chondrogenic differentiation potentials, of IFP-derived DFAT cells and IFP-derived MSCs.

## 2 Methods

### 2.1 Isolation of IFP-derived MSCs and DFAT cells

This study was approved by the Medical Research Ethics Committee of Tokyo Medical and Dental University, and informed consent was obtained from all study subjects. Human IFP was harvested from the knees of 8 OA donors during total knee arthroplasty.

**Fig. 1:**
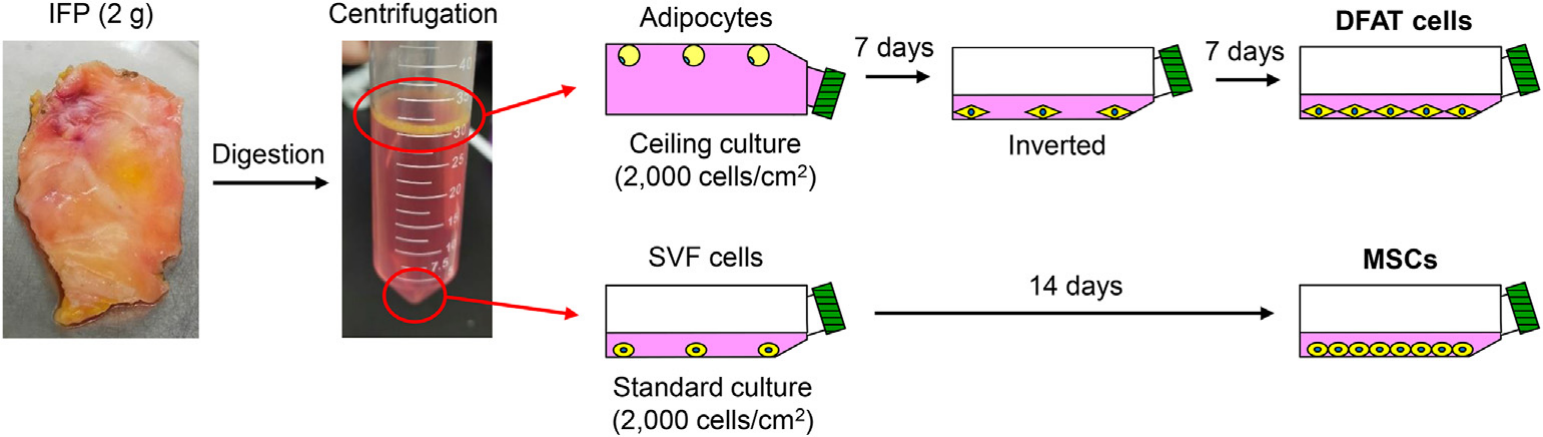
Schematic illustration of the study design. Approximately 2 g of IFP was digested and centrifuged. The floating top layer that contained mature adipocytes and the precipitate that contained SVF cells were collected. The isolated adipocytes were cultured in the ceiling of the flasks filled with growth medium. After 7 days of culture, the flasks were inverted and cultured for an additional 7 days. These cells were used as DFAT cells. MSCs were obtained by expanding SVF cells in the standard culture for 14 days.

Approximately 2 g of IFP was minced and digested in a solution of 3 mg/mL collagenase (Sigma-Aldrich Japan, Tokyo, Japan) at 37 °C for 1.5 h, and the digested cells were filtered through a 70 μm cell strainer (Greiner Bio-One GmbH, Frickenhausen, Germany). After centrifugation at 500×*g* for 5 min, the floating top layer that contained mature adipocytes was collected and washed with phosphate-buffered saline (PBS). The precipitate was collected as stromal vascular fraction (SVF) and washed with PBS. Adipocytes were subjected to ceiling culture at a density of 2,000 cells/cm^2^ in 75 cm^2^ flasks completely filled with growth medium consisting of α-minimum essential medium (Thermo Fisher Scientific, Rockford, IL, USA), 1% antibiotic-antimycotic (Thermo Fisher Scientific), and 20% fetal bovine serum (FBS; Thermo Fisher Scientific). After 7 days, the flask was inverted, and the culture was maintained with 15 mL growth medium for 7 additional days. These cells were regarded as DFAT cells. SVF cells were cultured at a density of 2,000 cells/cm^2^ in 75 cm^2^ flasks with 15 mL growth medium for 14 days. These cells were regarded as MSCs. After 14 days, both groups of cells were detached with 0.25% trypsin and 1 mM ethylenediaminetetraacetic acid (EDTA; Thermo Fisher Scientific). Cells at passage 2 were used for all experiments. An experimental scheme is shown in Fig. 1.

### 2.2 Flow cytometry

DFAT cells and MSCs were detached with TrypLE (Thermo Fisher Scientific) and suspended in PBS supplemented with 0.2% FBS and 5 mM EDTA at a density of 5 × 10^5^ cells/mL. The cells were stained for 30 min with the following antibodies: CD44 (PE-Cy7), CD45 (APC-H7), CD73 (V450), CD90 (PE), CD105 (APC), CD29 (APC), CD31 (PE-Cy7), CD34 (PE), CD146 (FITC), CD271 (PE-Cy7), CD140a (V450), and CD140b (PerCP-Cy5.5) (all from Becton Dickinson [BD], Franklin Lakes, NJ, USA). Cell fluorescence was evaluated using a FACSVerse instrument (BD). The data were analyzed using FlowJo software (Tree Star Software, Ashland, CA, USA).

### 2.3 Colony-forming assays

Cells were seeded at 100 cells/dish on a 60 cm^2^ (Thermo Fisher Scientific) dish and cultured for 14 days in growth medium. The cells were fixed with 10% neutral buffered formalin (Muto Pure Chemicals, Tokyo, Japan) and stained with 0.5% crystal violet (Wako, Osaka, Japan). Colonies were counted manually, and those with less than 2 mm in diameter were ignored.

### 2.4 Differentiation assays

For adipogenesis, 100 cells were cultured in growth medium in a 60 cm^2^ dish for 14 days to form cell colonies. The adherent cells were cultured for another 14 days in adipogenic induction medium consisting of growth medium supplemented with 100 nM dexamethasone (Wako), 0.5 mM isobutylmethylxanthine (Sigma-Aldrich), 50 mM indomethacin (Wako), 4.5 mg/mL D-(+)-glucose (Wako), and 10 μg/mL recombinant human insulin (Wako). The cells were stained with oil red O (Muto Pure Chemicals), and oil red O-positive colonies were counted. The stain was then extracted with isopropanol (Wako), and the optical density was measured at 510 nm with a microplate reader (Infinite M200; Tecan, Männedorf, Switzerland). Lastly, the colonies were stained with crystal violet for counting.

For osteogenesis, 100 cells were cultured in growth medium in a 60 cm^2^ dish for 14 days to form colonies. The adherent cells were cultured for an additional 14 days in osteogenic induction medium consisting of growth medium supplemented with 50 μg/mL ascorbate-2-phosphate (Sigma-Aldrich), 1 nM dexamethasone, and 10 mM β-glycerophosphate (Sigma-Aldrich). Calcification was assessed by alizarin red staining (Merck Millipore, Billerica, MA, USA), and alizarin red-positive colonies were counted. The stain was then extracted with 10% cetylpyridinium chloride (CPC; Wako) in 10 mM Na_2_PO_4_ (pH 7.0), and the optical density was measured at 560 nm with a microplate reader. Lastly, the colonies were stained with crystal violet for counting.

Chondrogenesis was examined by suspending 2.5 × 10^5^ cells in 0.5 mL chondrogenic induction medium consisting of Dulbecco's modified Eagle medium (Thermo Fisher Scientific) supplemented with 10 ng/mL transforming growth factor-β3 (Miltenyi Biotec, Bergisch Gladbach, Germany), 500 ng/mL bone morphogenetic protein 2 (Medtronic, Minneapolis, MN, USA), 40 μg/mL proline, 100 nM dexamethasone, 100 μg/mL pyruvate, 50 μg/mL ascorbate-2-phosphate, and 1% ITS Premix (BD). The cells were centrifuged at 500×*g* for 10 min and then cultured for 21 days. After 21 days of chondrogenic culture, 6 pellets in each group were weighted. Three pellets in each group were sectioned and stained with safranin O (Wako).

### 2.5 Biochemical analysis

For quantification of glycosaminoglycan (GAG) and DNA, chondrogenic pellets were digested with 100 μg/mL papain (Sigma-Aldrich) at 65 °C for 16 h. DNA content was measured by Hoechst 33342 dye staining (Dojindo, Tokyo, Japan). Fluorescence intensity was measured with a microplate reader (Tecan) at an excitation wavelength of 360 nm and an emission wavelength of 465 nm. Calf thymus DNA (Sigma-Aldrich) was used to generate a standard curve. The GAG content was quantified by a Blyscan Kit (Biocolor, Carrickfergus, UK) according to the manufacturer's instructions. The absorbance was measured at 656 nm to calculate the total GAG content. For comparison of GAG-producing ability, GAG content was normalized to DNA content (GAG/DNA).

### 2.6 RNA isolation and real-time quantitative PCR

After adipogenic, osteogenic, and chondrogenic differentiations, total RNA was extracted using miRNeasy Mini Kit (Qiagen, Hilden, Germany). Complementary DNA was generated with the Transcriptor High Fidelity cDNA Synthesis Kit (Roche Diagnostics, Basel, Switzerland). The real-time fluorescent intensity of SYBR Green dye (THUNDERBIRD Next SYBR qPCR Mix; Toyobo, Osaka, Japan) was monitored on the LightCycler 480 instrument (Roche Diagnostics, Basel, Switzerland). The following primers were used in this study: 18S ribosomal RNA, 5′-CACGGGTGACGGGGAATCAG-3′ (forward) and 5′-CGGGTCGGGAGTGGGTAATTTG-3′ (reverse); PPARG, 5′-GGATTCAGCTGGTCGATATCAC-3′ (forward) and 5′-GTTTCAGAAATGCCTTGCAGT-3′ (reverse); CEBPA, 5′-CCCTCCACCTTCATGTAGAAC-3′ (forward) and 5′-CCACGCCTGTCCTTAGAAAG-3′ (reverse); FABP4, 5′-ATCACATCCCCATTCACACT-3′ (forward) and 5′-ACTTGTCTCCAGTGAAAACTTTG-3′ (reverse); ALPL, 5′-TCCCTGATGTTATGCATGAGC-3′ (forward) and 5′-CGAGAGTGAACCATGCCA-3′ (reverse); COL1A1, 5′-TTCTGTACGCAGGTGATTGG-3′ (forward) and 5′-GACATGTTCAGCTTTGTGGAC-3′ (reverse); SOX9, 5′-CGTTCTTCACCGACTTCCTC-3′ (forward) and 5′-CTGGGCAAGCTCTGGAG-3′ (reverse); ACAN, 5′-AGATTCACAGAACTCCAGTGC-3′ (forward) and 5′-ACCTACGATGTCTACTGCTTTG-3′ (reverse); COL2A1, 5′-GTTTTCCAGCTTCACCATCATC-3′ (forward) and 5′-CCTCAAGGATTTCAAGGCAAT-3′ (reverse); COL10A1, 5′-GTACCTTGCTCTCCTCTTACTG-3′ (forward) and 5′-CATAAAAGGCCCACTACCCA-3′ (reverse). The PCR cycling conditions were as follows: 95 °C for 1 min, 45 cycles at 95 °C for 15 s, and 60 °C for 30–45 sec. 18S ribosomal RNA was used as an internal control, and each experiment was performed in duplicate.

### 2.7 Statistical analysis

Paired and unpaired Student's *t*-tests were used to compare the two groups. Data were expressed as the average ± standard deviation (SD). P values < 0.05 were considered statistically significant. All statistical analyses were performed using GraphPad Prism 6 (GraphPad Software, CA, USA).

## 3 Results

### 3.1 Morphology and cell yields of MSCs and DFAT cells

**Fig. 2:**
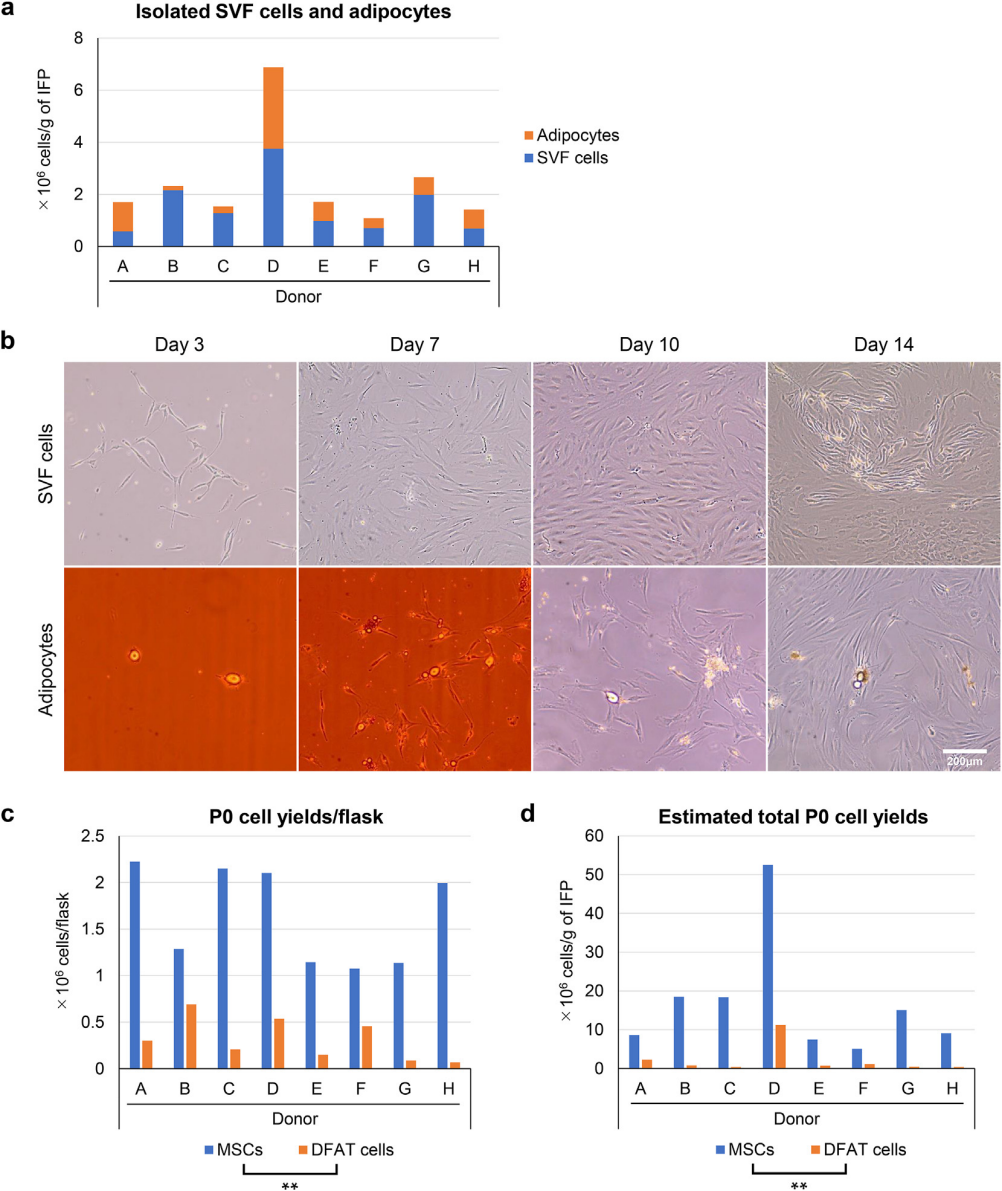
Morphology and cell yields. (a) Number of SVF cells and adipocytes isolated from 1 g of IFP. (b) Morphological changes in SVF cells and adipocytes. (c) Number of P0 cells obtained from one flask. (d) Estimated total P0 cell yields from 1 g of IFP. ∗∗p < 0.005, paired *t*-test.

The number of SVF cells and adipocytes isolated from 1 g of IFP is shown in Fig. 2a. The number of adipocytes was greater than the number of SVF cells in 2 of the 8 donors, while the opposite result was observed in the other 6 donors. Morphological changes in the IFP-derived SVF cells and adipocytes were observed over time (Fig. 2b). The SVF cells showed fibroblast-like morphologies at day 3 and rapidly proliferated to reach confluency on day 14. In the ceiling culture, some adipocytes attached to the ceiling of the flask and started to adopt an elongated shape on day 3. Fibroblast-like cells proliferated around the adipocytes on day 7 but had not yet reached confluency on day 14. The number of P0 cells obtained per flask was significantly greater in MSCs than in DFAT cells (Fig. 2c). The estimated total P0 cell yields obtained from 1 g of IFP were also significantly greater in MSCs than in DFAT cells (Fig. 2c). In all subsequent experiments, we used the cells from 4 randomly selected donors who had sufficient yields of both MSCs and DFAT cells.

### 3.2 Surface antigen profiles of MSCs and DFAT cells

**Fig. 3:**
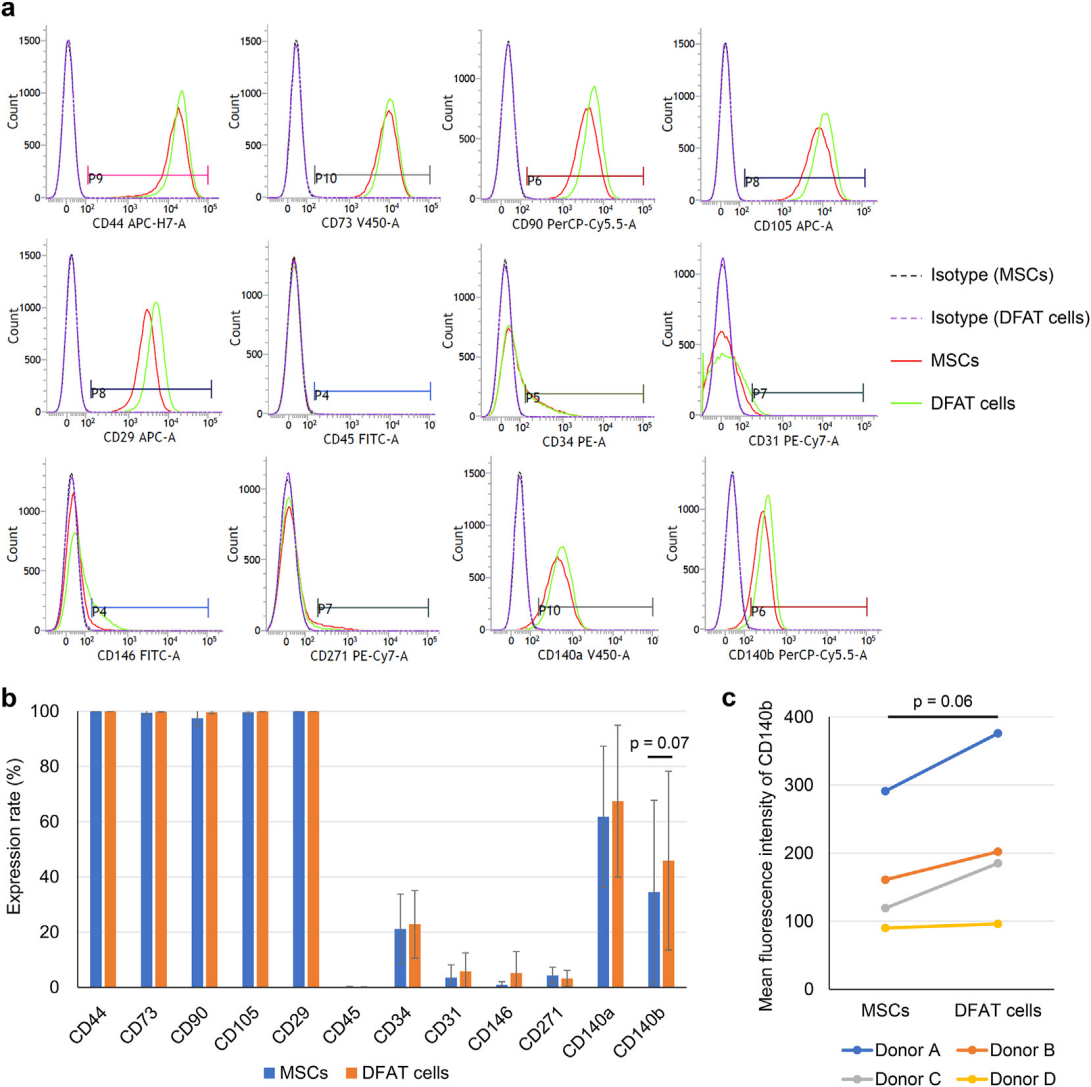
Surface antigen expressions. (a) Representative flow cytometry histograms and gates. The solid red lines indicate MSCs, and the solid green lines indicate DFAT cells. The dotted lines indicate isotype control. (b) Expression rate of surface antigens in MSCs and DFAT cells. Data are presented as mean ± SD from 4 donors (donor A-D). (c) Mean fluorescence intensity of CD140b in MSCs and DFAT cells. Statistical analysis was performed using a paired *t*-test.

The expressions of MSC markers (CD44, CD73, CD90, CD105, CD29), hematopoietic markers (CD45, CD34), endothelial cell markers (CD31, CD146), and markers used for MSC purification (CD271 [16], CD140a [17], CD140b [18]) were evaluated using flow cytometry (Fig. 3a). Both MSCs and DFAT cells expressed CD44, CD73, CD90, and CD105 at a rate of nearly 100% (Fig. 3b). The positive rate for CD140b was higher in DFAT cells, but not significantly (p = 0.07). The mean fluorescence intensity of CD140b also increased in DFAT cells, but not significantly (Fig. 3c, p = 0.06). No remarkable differences were found between MSCs and DFAT cells in the expression of the other markers.

### 3.3 Colony-forming ability

**Fig. 4:**
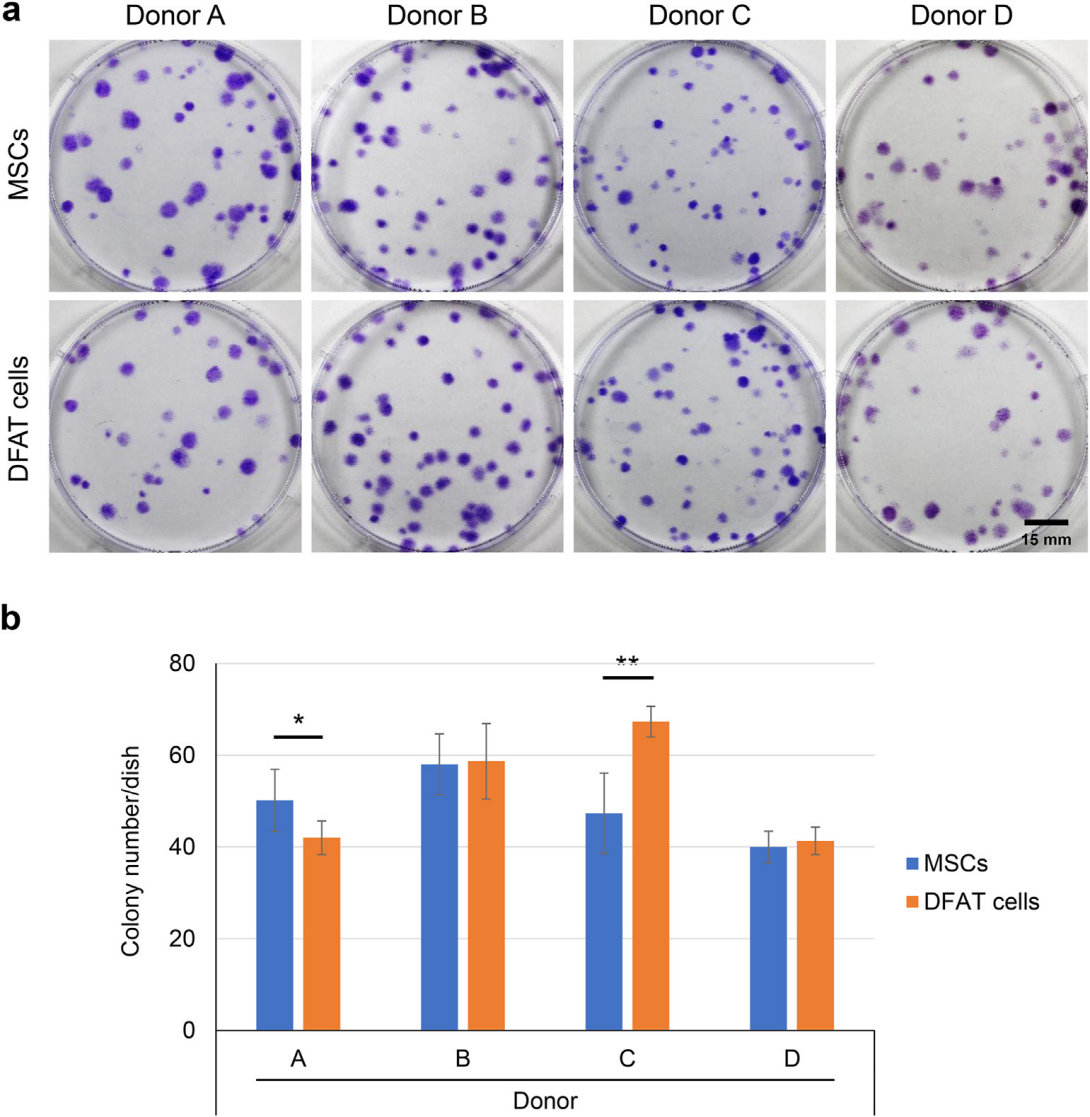
Colony-forming ability. (a) Representative images of colonies stained with crystal violet. (b) Colony number per dish. Data are presented as mean ± SD (6 replicate dishes per donor). ∗p < 0.05, ∗∗p < 0.005, unpaired *t*-test.

One hundred cells were cultured for 14 days, and the formed colonies were stained with crystal violet (Fig. 4a). For donor A, the colony number was significantly greater in MSCs than in DFAT cells (Fig. 4b). For donor C, the opposite result was obtained. No significant differences between the MSCs and DFAT cells were observed for the other donors.

### 3.4 Adipogenic ability

**Fig. 5:**
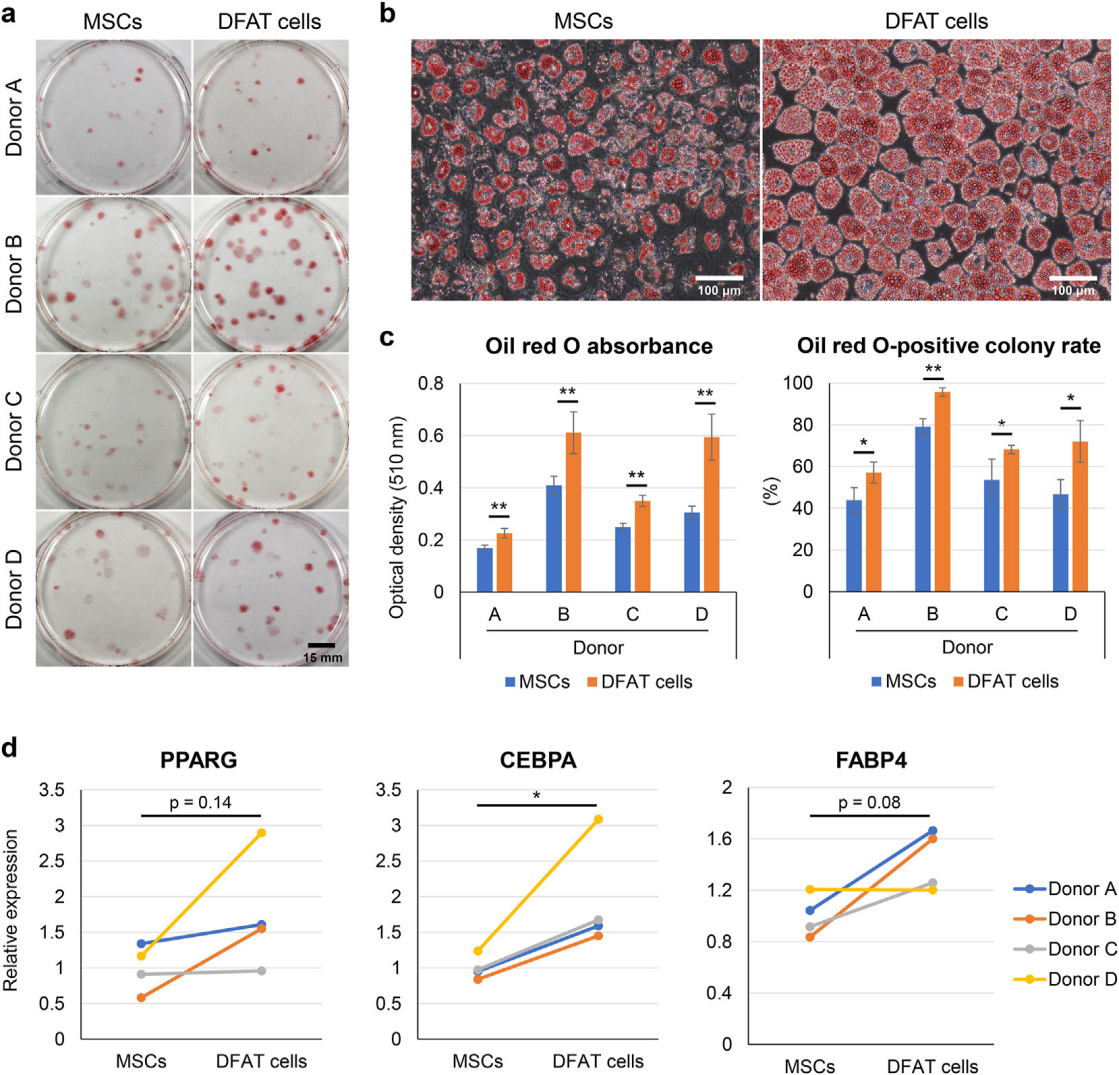
Adipogenic ability. (a) Representative images of oil red O staining. (b) Representative microscopic images. (c) Oil red O absorbance and oil red O-positive colony rate. Data are presented as mean ± SD (4 replicate dishes per donor). (d) Relative mRNA expression of adipogenic markers. ∗p < 0.05, ∗∗p < 0.005, unpaired *t*-test.

We cultured the MSCs and DFAT cells in growth medium for 14 days to form colonies. We then cultured them in adipogenic induction medium for 14 days and stained them with oil red O (Fig. 5a). Under microscopic observation, the DFAT cells had more lipid droplets positive for oil red O than the MSCs did (Fig. 5b). Oil red O was dissolved in isopropanol, and the absorbance was quantitatively evaluated (Fig. 5c). The optical density value of the oil red O stain was significantly higher in the DFAT cells than in the MSCs for all donors. All colonies were stained with crystal violet to calculate the percentage of oil red O-stained colonies. The DFAT cells showed a significantly higher oil red O-positive colony rate than the MSCs for all donors. Real-time PCR analysis showed a significant upregulation of CEBPA expression in the DFAT cells (Fig. 5d). The expression of PPARG and FABP4 was higher in the DFAT cells, but not significantly (p = 0.14 and 0.08, respectively).

### 3.5 Osteogenic ability

**Fig. 6:**
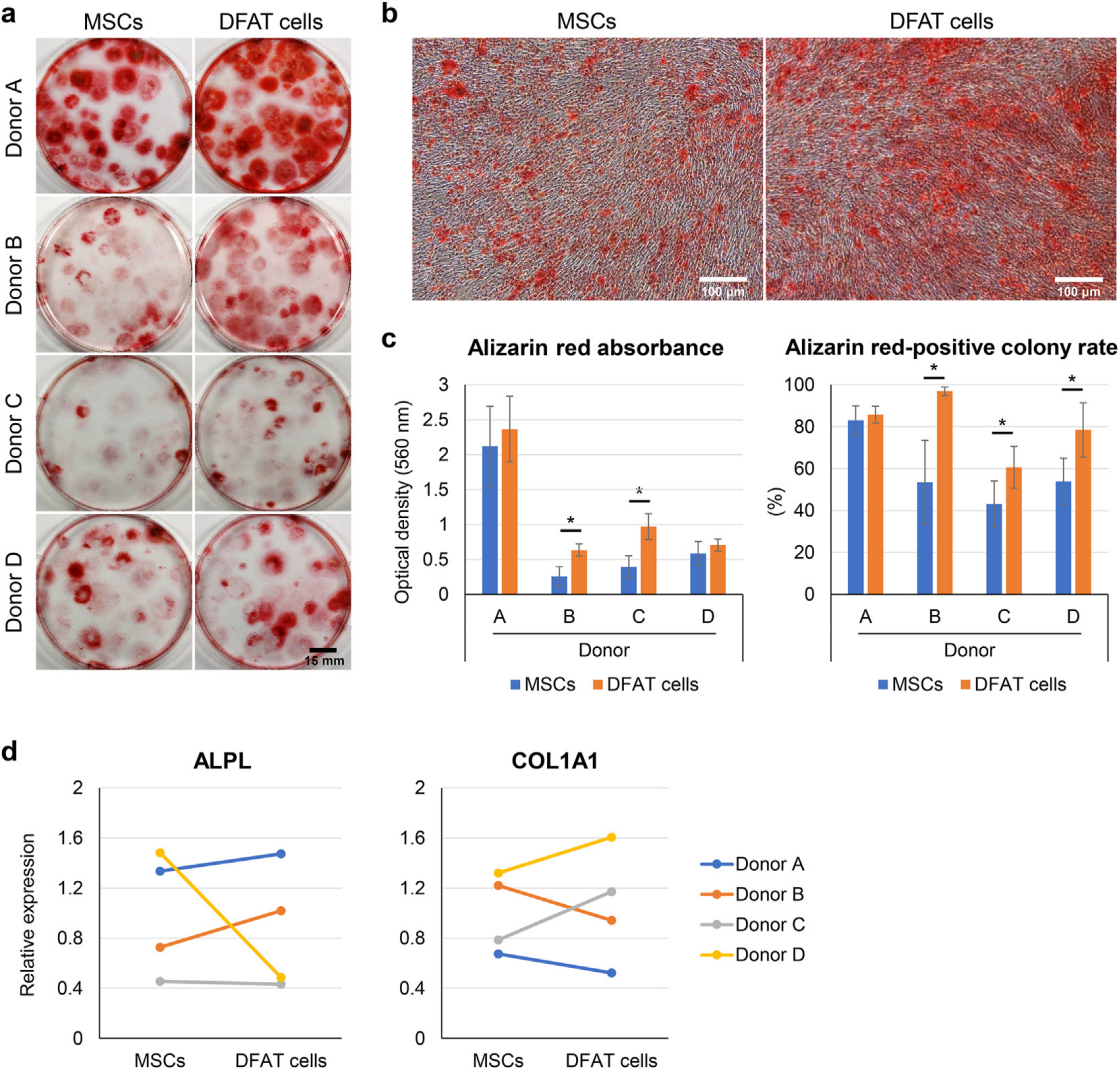
Osteogenic ability. (a) Representative images of alizarin red staining. (b) Representative microscopic images. (c) Alizarin red absorbance and alizarin red-positive colony rate. Data are presented as mean ± SD (4 replicate dishes per donor). (d) Relative mRNA expression of osteogenic markers. ∗p < 0.05, unpaired *t*-test.

We cultured the MSCs and DFAT cells in growth medium for 14 days to form colonies. We then cultured them in osteogenic induction medium for 14 days and stained them with alizarin red (Fig. 6a). Under microscopic observation, the DFAT cells were more strongly stained with alizarin red than the MSCs were (Fig. 6b). Alizarin red was dissolved in 10% CPC, and the absorbance was quantitatively evaluated (Fig. 6c). The optical density value of alizarin red was significantly higher in the DFAT cells than in the MSCs for 2 of the 4 donors. All colonies were stained with crystal violet to calculate the percentage of alizarin red-stained colonies. The DFAT cells showed a significantly higher alizarin red-positive colony rate than the MSCs did for 3 of the 4 donors. The expression of osteogenic genes (ALPL and COL1A1) did not reveal a remarkable difference between the MSCs and DFAT cells (Fig. 6d).

### 3.6 Chondrogenic ability

**Fig. 7:**
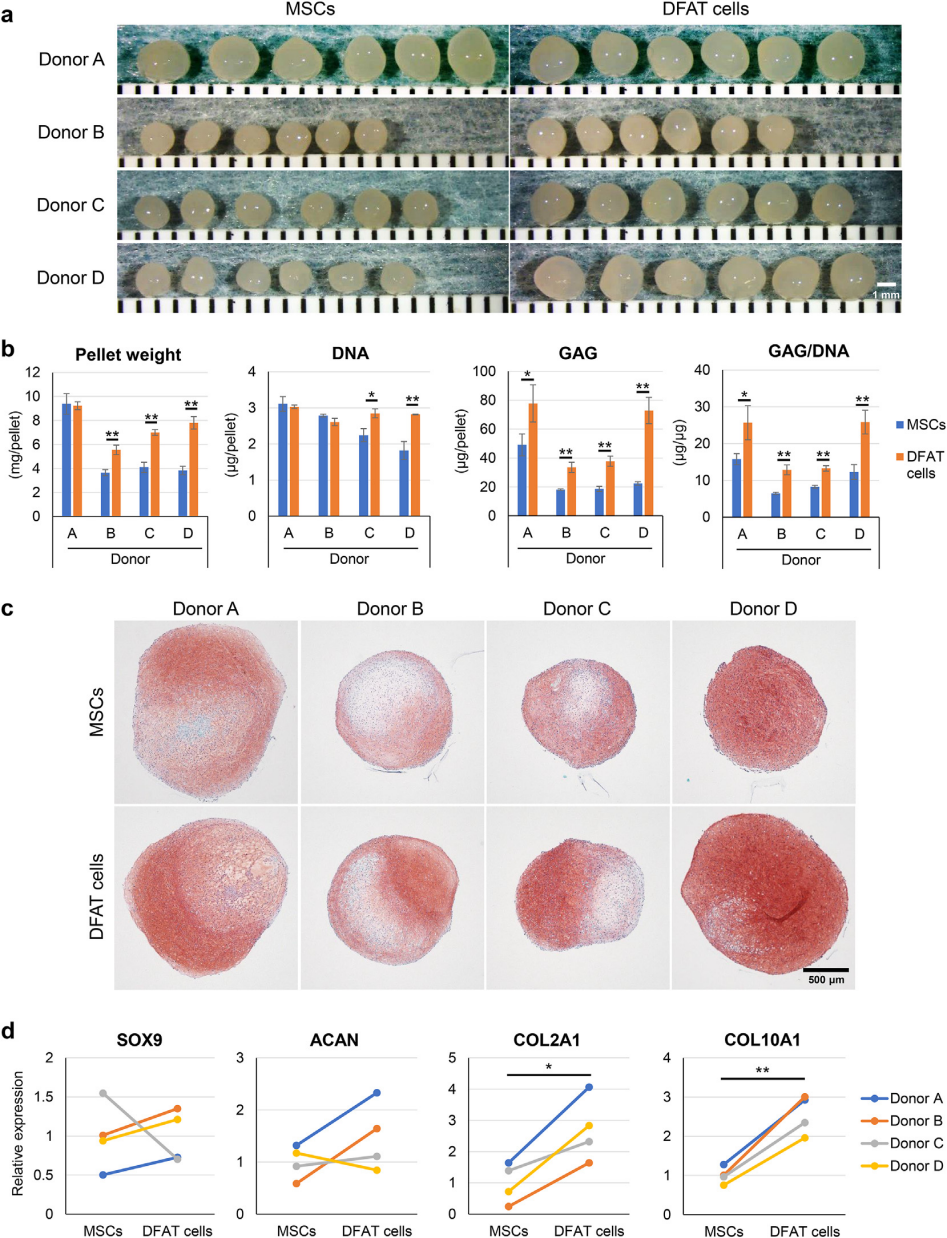
Chondrogenic ability. (a) Gross appearance of chondrogenic pellets (6 replicate pellets per donor). (b) Pellet wet weight, DNA amount, glycosaminoglycan (GAG) amount, and GAG/DNA ratio. Data are presented as mean ± SD (3 replicate pellets per donor). (c) Representative images of chondrogenic pellets stained with safranin O. (d) Relative mRNA expression of chondrogenic markers. ∗p < 0.05, ∗∗p < 0.005, unpaired *t*-test.

Cell pellets were cultured in chondrogenic induction medium for 21 days to evaluate chondrogenic potential. The DFAT cells formed larger pellets than the MSCs did (Fig. 7a). Pellet weight was significantly greater in the DFAT cells than in the MSCs for 3 of the 4 donors (Fig. 7b). The DNA amount of the DFAT cells was significantly greater than that of the MSCs for 2 of the 4 donors. The GAG amount and GAG/DNA ratio were significantly greater in the DFAT cells than in the MSCs for all donors. The pellets derived from DFAT cells were more intensely stained with safranin O than those from the MSCs (Fig. 7c). Although SOX9 and ACAN showed similar expression levels in the MSCs and DFAT cells, the expression of COL2A1 and COL10A1 was significantly upregulated in the DFAT cells (Fig. 7d).

## 4 Discussion

The number of SVF cells isolated from the IFPs of OA patients was greater than that of mature adipocytes in 6 out of 8 patients, and the estimated total P0 cell yield from 1 g of IFP was much greater in the MSCs than in the DFAT cells. Compared to SC, IFP contains more fibrous tissue and has a higher specific gravity, especially in elderly donors [8], and IFP fibrosis is often found in OA knees [19]. These findings suggest that the IFP from OA patients is composed of fewer adipocytes and more SVF cells than SC. Matsumoto et al. reported that 3 × 10^7^ DFAT cells could be obtained from 5 × 10^4^ of mature adipocytes of SC [12]. In this study, we used 1.5 × 10^5^ adipocytes per flask, but the resulting P0 yield of the DFAT cells was less than 1 × 10^6^ cells per flask. Given that IFP-derived DFAT cells are reported to have greater proliferative potential than SC-derived DFAT cells [15], the dedifferentiation ratio of IFP-derived adipocytes may be much lower than that of SC-derived adipocytes.

In our study, the IFP-derived adipocytes that adhered to the tops of plastic flasks changed their morphology to an elongated shape and then started to proliferate. These cells were positive for CD105, CD73, and CD90, were negative for CD45, and showed colony formation and trilineage differentiation abilities. Therefore, the IFP-derived DFAT cells we prepared in this study met the minimal criteria of MSCs [20].

Flow cytometric analysis revealed that the IFP-derived MSCs and DFAT cells had similar expressions of MSC negative and positive markers. This finding is consistent with a study by Figiel-Dabrowsk et al., which showed no differences in surface antigen expression between SC-derived MSCs and DFAT cells [21]. However, some studies found that DFAT cells expressed higher levels of CD44 and lower levels of CD45 and CD31 than MSCs did [12,22]. In contrast, we found that IFP-derived DFAT cells tended to express a greater level of CD140b than IFP-derived MSCs did. CD140b is also a platelet-derived growth factor (PDGF) receptor β, and it is used for identifying MSCs with greater abilities [18]. According to Uomizu et al., PDGF signaling plays an important role in the proliferation and differentiation of human synovial MSCs [23], and the higher level of CD140b expression in IFP-derived DFAT cells may contribute to their greater differentiation potentials.

In our study, the IFP-derived DFAT cells exhibited higher adipogenic ability than the IFP-derived MSCs did. Previous studies comparing SC-derived MSCs and DFAT cells have shown similar results [13,14,24]. Therefore, DFAT cells should have greater adipogenic potential than MSCs, regardless of the collection site.

Regarding chondrogenesis, the IFP-derived DFAT cells produced a significantly greater amount of GAG, resulting in the formation of heavier pellets strongly stained with safranin O. Quantitative PCR analysis revealed a higher expression of chondrogenic genes in DFAT cells. These findings indicate that IFP-derived DFAT cells have better chondrogenic differentiation ability than IFP-derived MSCs. Mochizuki et al. previously reported that IFP-derived MSCs have as high a chondrogenic potential as fibrous synovium-derived MSCs [8]. Consequently, IFP-derived DFAT cells could be one of the most ideal cell sources for cartilage regenerative medicine. However, the number of DFAT cells that we were able to collect from IFP was very low compared to the number MSCs we could collect from the same weight of IFP. For the clinical use of IFP-derived DFAT cells, the dedifferentiation rate must be improved. Li et al. recently demonstrated that physical compression could induce the dedifferentiation of adipocytes via activating Wnt/β-catenin signaling [25].

This study had three main limitations. First, we used IFP obtained from OA patients who underwent total knee arthroplasty. The characteristics of DFAT from healthy IFPs remain unclear. Second, we used P2 cells to compare the properties, as the amount of IFP-derived DFAT cells obtained at P0 was very small. Given that prolonged in vitro expansion is known to change MSC properties [26], P0 cells might have different results. Third, we did not perform in vivo experiments. To clarify whether IFP-derived MSCs or DFAT cells have a greater ability to repair cartilage, the transplantation of these cells into cartilage defects will be necessary.

## 5 Conclusions

The adipocytes derived from IFP dedifferentiated during ceiling culture and acquired stem cell properties. Compared with the IFP-derived MSCs, the IFP-derived DFAT cells were superior in adipogenic and chondrogenic abilities but had a poor cell yield. Our findings indicate that IFP-derived DFAT cells appeared to be a useful cell source for cartilage regenerative medicine if we can increase their yield. Further studies are needed to assess their in vivo chondrogenic potential.

## Ethics approval and consent to participate

This study was approved by the Medical Research Ethics Committee of Tokyo Medical and Dental University. Written informed consent forms were obtained from all participants.

## Consent for publication

Not applicable.

## Availability of data and materials

The datasets used and/or analyzed during the current study are available from the corresponding author upon reasonable request.

## Declaration of competing interests

The authors declare that they have no known competing financial interests or personal relationships that could have appeared to influence the work reported in this paper.

## Funding

This study was supported by 10.13039/501100001691JSPS KAKENHI Grant Number 20K17990 (author KE) and by the 10.13039/100009619Japan Agency for Medical Research and Development under Grant Number JP21bk0104103 (author IS).

## Author contributions

SF provided ideas, performed all experiments, analyzed and interpreted the data, and drafted the manuscript. KE designed the study, provided ideas, organized the data, and completed the manuscript. SM contributed to the acquisition of PCR data and revised the manuscript. KK analyzed the data and revised the manuscript. IS provided ideas and revised the manuscript. All authors read and approved the final manuscript.

## Declaration of Competing Interest

The authors declare that they have no conflicts of interest.

## References

[1] Van der Kraan P.M., Buma P., Van Kuppevelt T., Van Den Berg W.B. (2002). Interaction of chondrocytes, extracellular matrix and growth factors: relevance for articular cartilage tissue engineering. Osteoarthritis Cartilage.

[2] Alford J.W., Cole B.J. (2005). Cartilage restoration, part 1: basic science, historical perspective, patient evaluation, and treatment options. Am J Sports Med.

[3] Harris J.D., Siston R.A., Pan X., Flanigan D.C. (2010). Autologous chondrocyte implantation: a systematic review. J Bone Jt Surg - Ser A.

[4] Ding D.-C., Shyu W.-C., Lin S.-Z. (2011). Mesenchymal stem cells. Cell Transplant.

[5] De Ugarte D.A., Morizono K., Elbarbary A., Alfonso Z., Zuk P.A., Zhu M. (2003). Comparison of multi-lineage cells from human adipose tissue and bone marrow. Cells Tissues Organs.

[6] Sakaguchi Y., Sekiya I., Yagishita K., Muneta T. (2005). Comparison of human stem cells derived from various mesenchymal tissues: superiority of synovium as a cell source. Arthritis Rheum.

[7] Yao B., Samuel L.T., Acuña A.J., Faour M., Roth A., Kamath A.F. (2021). Infrapatellar fat pad resection or preservation during total knee arthroplasty: a systematic review. J Knee Surg.

[8] Mochizuki T., Muneta T., Sakaguchi Y., Nimura A., Yokoyama A., Koga H. (2006). Higher chondrogenic potential of fibrous synovium- and adipose synovium-derived cells compared with subcutaneous fat-derived cells: distinguishing properties of mesenchymal stem cells in humans. Arthritis Rheum.

[9] Francis S.L., Yao A., Choong P.F.M. (2020).

[10] Sugihara H., Funatsumaru S., Yonemitsu N., Miyabara S., Toda S., Hikichi Y. (1989). A simple culture method of fat cells from mature fat tissue fragments. J Lipid Res.

[11] Yagi K., Kondo D., Okazaki Y., Kano K. (2004). A novel preadipocyte cell line established from mouse adult mature adipocytes. Biochem Biophys Res Commun.

[12] Matsumoto T., Kano K., Kondo D., Fukuda N., Iribe Y., Tanaka N. (2008). Mature adipocyte-derived dedifferentiated fat cells exhibit multilineage potential. J Cell Physiol.

[13] Saler M., Caliogna L., Botta L., Benazzo F., Riva F., Gastaldi G. (2017). hASC and DFAT, multipotent stem cells for regenerative medicine: a comparison of their potential differentiation in vitro. Int J Mol Sci.

[14] Fujisaki S., Kajiya H., Yanagi T., Maeshiba M., Kakura K., Kido H. (2021). Enhancement of jaw bone regeneration via ERK1/2 activation using dedifferentiated fat cells. Cytotherapy.

[15] Tanimoto K., Matsumoto T., Nagaoka Y., Kazama T., Yamamoto C., Kano K. (2022). Phenotypic and functional properties of dedifferentiated fat cells derived from infrapatellar fat pad. Regen Ther.

[16] Álvarez-Viejo M., Menéndez-Menéndez Y., Otero-Hernández J. (2015). CD271 as a marker to identify mesenchymal stem cells from diverse sources before culture. World J Stem Cell.

[17] Li H., Ghazanfari R., Zacharaki D., Ditzel N., Isern J., Ekblom M. (2014). Low/negative expression of PDGFR-α identifies the candidate primary mesenchymal stromal cells in adult human bone marrow. Stem Cell Rep.

[18] Wang S., Mo M., Wang J., Sadia S., Shi B., Fu X. (2018). Platelet-derived growth factor receptor beta identifies mesenchymal stem cells with enhanced engraftment to tissue injury and pro-angiogenic property. Cell Mol Life Sci.

[19] Favero M., El-Hadi H., Belluzzi E., Granzotto M., Porzionato A., Sarasin G. (2017). Infrapatellar fat pad features in osteoarthritis: a histopathological and molecular study. Rheumatology.

[20] Dominici M., Le Blanc K., Mueller I., Slaper-Cortenbach I., Marini F.C., Krause D.S. (2006). Minimal criteria for defining multipotent mesenchymal stromal cells. The International Society for Cellular Therapy position statement. Cytotherapy.

[21] Figiel-Dabrowska A., Radoszkiewicz K., Rybkowska P., Krzesniak N.E., Sulejczak D., Sarnowska A. (2021). Neurogenic and neuroprotective potential of stem/stromal cells derived from adipose tissue. Cells.

[22] Tansriratanawong K., Tabei I., Ishikawa H., Ohyama A., Toyomura J., Sato S. (2020). Characterization and comparative DNA methylation profiling of four adipogenic genes in adipose-derived stem cells and dedifferentiated fat cells from aging subjects. Hum Cell.

[23] Uomizu M., Muneta T., Ojima M., Sekiya I., Koga H., Tsuji K. (2018). PDGF-induced proliferation and differentiation of synovial mesenchymal stem cells is mediated by the PI3K-PKB/Akt pathway. J Med Dent Sci.

[24] Nobusue H., Kano K. (2010). Establishment and characteristics of porcine preadipocyte cell lines derived from mature adipocytes. J Cell Biochem.

[25] Li Y., Mao A.S., Seo B.R., Zhao X., Gupta S.K., Chen M. (2020). Compression-induced dedifferentiation of adipocytes promotes tumor progression. Sci Adv.

[26] Jeske R., Yuan X., Fu Q., Bunnell B.A., Logan T.M., Li Y. (2021). In Vitro culture expansion shifts the immune phenotype of human adipose-derived mesenchymal stem cells. Front Immunol.

